# Identifying Pertinent Digital Health Topics to Incorporate into Self-Care Pharmacy Education

**DOI:** 10.3390/pharmacy12030096

**Published:** 2024-06-20

**Authors:** Jason C. Wong, Luiza Hekimyan, Francheska Anne Cruz, Taylor Brower

**Affiliations:** 1 Pharmacy Practice and Administration, Western University of Health Sciences, Pomona, CA 91766, USA; 2 College of Pharmacy, Western University of Health Sciences, Pomona, CA 91766, USA; luiza.hekimyan@westernu.edu (L.H.); francheskaanne.cruz@westernu.edu (F.A.C.); taylor.brower@westernu.edu (T.B.)

**Keywords:** applications, devices, digital health, education, self-care, technology, pharmacy students

## Abstract

The ever-evolving landscape of digital health technology has dramatically enhanced patients’ ability to manage their health through self-care effectively. These advancements have created various categories of self-care products, including medication management, health tracking, and wellness. There is no published research regarding integrating digital health into pharmacy self-care courses. This study aims to identify pertinent digital health devices and applications to incorporate into self-care course education. Digital health limitations, challenges incorporating digital health in self-care pharmacy education, and potential solutions are also reviewed. In conducting this research, many resources, including PubMed, APhA, ASHP, fda.gov, and digital.health, were reviewed in March 2024 to gather information on digital health devices and applications. To supplement this, targeted keyword searches were conducted on topics such as “digital health”, “devices”, “applications”, “technology”, and “self-care” across various online platforms. We identified digital health devices and applications suitable for self-care education across eight topics, as follows: screening, insomnia, reproductive disorders, eye disorders, home medical equipment, GI disorders, pediatrics, and respiratory disorders. Among these topics, wellness screening had the most digital health products available. For all other topics, at least three or more products were identified as relevant to self-care curriculum. By equipping students with digital health knowledge, they can effectively apply it in patient care throughout their rotations and future practice. Many digital health products, including telemedicine, electronic health records, mobile health applications, and wearable devices, are ideal for inclusion in pharmacy curriculum as future educational material. Future research is needed to develop the best strategies for incorporating relevant digital health into self-care education and defining the best student-learning strategies.

## 1. Introduction

Digital health encompasses technologies that help patients monitor and manage their medical conditions more efficiently. It offers tools, like a smartphone or a wearable sensor with medical applications, for accessing health information, tracking progress, and managing care remotely [1]. Patients can receive personalized attention, easily schedule appointments, attend virtual telehealth consultations, and be more engaged in their care, ultimately empowering them to control their health and improve their quality of life [2].

Digital health is transforming the way clinicians interact with patients and handle medicine by providing tools that allow for electronic prescribing, access to patient data, virtual monitoring, telehealth, support tools, virtual health information, patient education, efficiency and workflow, and continuous professional development [3,4]. This improves access to care and enhances clinical decision-making skills, ultimately optimizing healthcare delivery to patients. However, it is important to remember that digital health also has its drawbacks, such as privacy and security concerns [5,6].

It is increasingly important for healthcare providers to embrace digital health practices to improve patient care as technology continues to dominate modern life. This innovative approach, which harnesses the power of technology to diagnose and treat patients, has been recognized by the World Health Organization as a means of advancing global health and improving quality of life [7]. Various mobile applications and user-friendly medical devices are now available to help improve patients’ quality of life and life expectancy. Patients can find areas such as wellness and screening digital health trackers that allow them to independently check their vital signs and health conditions. Across the globe, there are examples of government surveillance in the digital health sector. For instance, the Australian government has established the Australian Digital Health Agency, which is a public online hub that integrates digital health services. These services include accessing personal digital health records, prescribing prescriptions electronically, and making telehealth appointments online [8]. From user-friendly mobile apps to cutting-edge medical devices, digital health technologies are revolutionizing patient care and helping to bridge the gap in access to healthcare for underserved communities [9].

Pharmacists play a crucial role as accessible healthcare providers, offering valuable support to patients seeking self-care solutions. They provide consultations, encourage medication adherence, offer nonpharmacological recommendations for medical conditions, and participate in public health initiatives such as smoking cessation, vaccination, and hypertension management [10]. Pharmacists need to adapt to digital health practices to serve patients better. For instance, they can use telehealth services to assist elderly patients with transportation difficulties. To ensure pharmacy students are well prepared, pharmacy schools should include digital health education in their curriculum, equipping students to effectively advise patients on using electronic medical devices and mobile applications [11].

With the rise of digital health, patients have greater access to tools to improve their quality of life. These tools, including mobile applications and electronic devices, can manage various health conditions in and out of clinical settings. The convenience of digital health empowers patients to take control of their health and strengthens their connection with their healthcare provider(s). Limited research has been conducted to determine suitable digital health education for self-care subjects. This paper presents an outline of pertinent digital health resources relating to self-care. It identifies those fit for incorporation into a pharmacy self-care program for student pharmacists. By acquiring proficiency in this knowledge, aspiring pharmacists can effectively assist patients in practicing self-care to prevent and treat common ailments.

## 2. Materials and Methods

Data were extracted online using the search term “digital health in self-care” on PubMed which revealed 4713 results across 472 pages. No additional software or tools were utilized. However, only five articles were relevant to our query. The data were independently double-checked by two co-authors. All authors agree with the data extraction process and its results.

We utilized the American Pharmacists Association (APhA) pharmacy library to supplement our research and access digital health resources, including practice tools, health organizations, and publications. Additional information on digital solutions that can improve patient experience through increased data access and sharing is retrieved from the American Society of Health-System Pharmacists (ASHP) website. Additionally, we explored various websites for relevant information and identified “digital.health”, “fda.gov”, and “dtxalliance.org” as the top three resources. The digital health products’ manufacturer websites provided detailed information on various devices, their applications, and corresponding accessories mentioned in this research paper.

### 2.1. Inclusion and Exclusion Criteria

The primary criteria for the inclusion of the applications and devices discussed in this article are their suitability for self-care, both for patients and their caregivers, as well as their relevance in the self-care education of pharmacy students. Other considerations included cost-effectiveness, FDA clearance, and devices that are currently in development. This ensures that pharmacy students are familiar with the devices and apps that can be used in patient care. FDA clearance indicates that the devices have been approved by the United States Food and Drug Administration as being equivalent to other legally marketed devices.

We excluded applications and devices that are only used in hospitals or ambulatory care clinics because they are not suitable for a self-care pharmacy course. Our search was limited to applications and devices available in English. We also excluded applications and devices that lacked sufficient information and data from the manufacturer’s website, not available for purchase or subscription, as well as those without information in English. Additionally, we excluded applications and devices that were considered too complex for layman-patient use after our validation process.

Our research findings can be replicated by other future researchers, as our investigation was carried out using publicly available data on the Internet. However, it is important to note that the details of digital health products may change in the future due to manufacturers’ updates.

### 2.2. Validity of Self-Care Digital Health Products/Applications

After applying the inclusion and exclusion criteria, 3 devices or applications were excluded and 29 were chosen for independent validation by a group of 13 individuals, comprising the authors, a licensed pharmacist, and other pharmacy students who had completed self-care courses.

**Figure 1:**
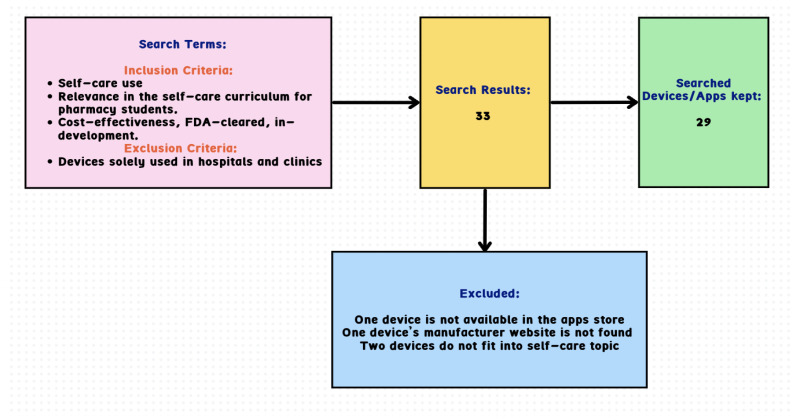
Inclusion/exclusion criteria for the devices and applications and the search results.

We performed a comprehensive validation process for the digital health devices and apps, classifying them into three groups. Firstly, we assessed whether the products/apps were technologically pertinent to self-care. Subsequently, we evaluated their alignment with existing pharmacy self-care curricula. Finally, we looked for evidence of educational enhancement provided by the products/apps, for either pharmacists or patients/caregivers. Each product/app received one point if it was considered relevant to self-care topics by the validator and zero points if not. If the product was compatible with existing self-care curricula, it received one point. Additionally, if there was evidence of educational enhancement for pharmacists or patients/caregivers, it received one point. Following this validation process, all except two of the digital health products were found to be suitable for use as self-care products in educational settings for pharmacy students. Refer to Figure 1 for the inclusion and exclusion criteria for the devices and applications and the search results.

## 3. Results

### 3.1. Self-Care Digital Health Products/Applications’ Validation

A comprehensive search of digital health products was conducted from multiple websites as part of the research to identify digital health topics for education in a self-care course.

Thirteen individuals independently rated the products and applications. Two first-year students, five second-year PharmD students, four third-year PharmD students, one pharmacist, and one pharmacy faculty participated in this validation.

Following the validation of these digital health products, all but two were found to be suitable for use as self-care products in educational settings for pharmacy students. See Table 1 for the validation results.

### 3.2. Summary of Digital Health Products/Applications Pertinent to Self-Care Education

The products mentioned above were categorized and grouped according to the various self-care topics they address. For a detailed breakdown of the products available under each self-care topic, refer to Table 2, Table 3, Table 4, Table 5, Table 6, Table 7, Table 8 and Table 9.

## 4. Discussion

### 4.1. Digital Health Devices and Applications

Within self-care courses, various digital health products—such as devices and system applications—can be utilized to aid in the learning process. Across different platforms, a number of tools have been identified in this research that are suitable for self-care courses, covering topics such as screening, insomnia, gastrointestinal (GI) disorders, reproductive disorders, eye disorders, pediatrics, respiratory disorders, home medical equipment, and applications compatible with both iOS and Android devices (Table 2, Table 3, Table 4, Table 5, Table 6, Table 7, Table 8 and Table 9). According to the World Health Organization (WHO), digital health encompasses numerous technologies and innovations, including health information technology, telehealth, health analytics, mobile health apps, digital therapeutics, artificial intelligence, blockchain, and robotics. These components work together to enhance healthcare systems and achieve the Sustainable Development Goals (SDGs) by catering to the diverse needs of individuals, healthcare providers, managers, and systems across the globe [40]. Digital health comprises a broad range of technologies designed to improve overall patient outcomes and efficiency within the healthcare system.

### 4.2. Incorporating Digital Health into Pharmacy Curriculum

#### 4.2.1. Digital Health and Self-Care Education

Previous studies have confirmed that digital health provides a promising opportunity to explore and implement self-care management, but we lack sufficient digital health education in the pharmacy curriculum. Research from Italy found that community pharmacists have insufficient knowledge of digital health, which may impede their ability to assist patients effectively. This deficiency could potentially pose challenges for patients seeking self-care assistance. Additionally, a study from Turkey revealed that more than 80% of pharmacy students believe that mobile health apps enhance the quality of life for their patients. However, approximately 30% feel pharmacists have inadequate digital health knowledge [41]. 

It is crucial to incorporate digital health education into the pharmacy curriculum. As stated in the article “Digital Health in Pharmacy Education: Preparedness and Responsiveness of Pharmacy Programs”, the author emphasizes that pharmacists must have a clear understanding of the fundamentals of digital health technologies and know how to integrate them into their clinical practices for both pharmacists and their patients to benefit from the potential advantages of digital health fully. Therefore, it is imperative to develop a digital health curriculum that focuses on building competencies to equip students with the necessary knowledge and skills to effectively practice digital health in their future professional careers [11].

However, digital health has not yet been fully integrated into the standard pharmacy curriculum because of its relative newness and constant evolution. It is, therefore, imperative to begin identifying relevant digital health topics and incorporating them into self-care education for pharmacy students. In the paragraphs below, we explore several devices and apps that are particularly pertinent to teaching self-care courses, organized by the relevant self-care topics.

In our program, self-care courses are scheduled during the fall and spring semesters of the first-year curriculum. During the fall semester, we focus on topics such as coughs and the common cold, gastrointestinal disorders, dermatologic conditions, and insomnia. The remaining self-care topics are covered during the spring semester. We can incorporate digital health education tailored to the body systems relevant to each self-care topic. The learning outcomes of our program related to digital health encompass providing patient-centered care, promoting health and wellness, addressing potential health issues, educating patients, and safeguarding the best interests of patients. We can employ effective learning strategies for digital health education, including team-based peer-teaching, preclass preparation (such asprimer readings before lectures), case discussions, or SOAP note applications, role-playing, and observed structured clinical examination (OSCE).

Regarding wellness screening, various digital health devices and apps can monitor blood pressure and heart rate, providing valuable insight into a patient’s well-being (refer to Table 2). Price, patient friendliness, and accuracy are important when evaluating these products or apps. Wellness screenings for blood pressure and EKG through digital health devices have become increasingly popular and convenient.

Various digital health devices and applications are available to help relieve insomnia and enhance sleep quality (refer to Table 3). These tools utilize different approaches, including sleep tracking, relaxation techniques, and cognitive behavioral therapies. Devices and apps for managing insomnia include those focused on sleep sounds, relaxation, and light therapy. These digital health solutions are specifically designed to aid individuals in addressing their sleep disturbances. As future pharmacists, it is essential to recognize the significance of sleep and to incorporate these resources into discussions on self-care. 

Digital health apps and devices focused on reproductive disorders are valuable resources for educating individuals dealing with fertility issues, menstrual irregularities, and other sexual health conditions (refer to Table 4). Many patients may feel uncomfortable or hesitant to discuss these sensitive issues with a healthcare provider. Including information about these resources in self-care courses is an effective way for pharmacy students to promote awareness of reproductive and sexual health early in their careers and improve patient access to these digital tools. 

Digital health devices and applications related to eye disorders are crucial in empowering individuals to preserve their eye health and maintain good vision (refer to Table 5). We can significantly contribute to improved vision outcomes and overall eye health by leveraging these tools. It is important to educate pharmacy students about their pivotal role in promoting self-care for eye disorders through activities such as patient education, early detection and monitoring, medication management, and virtual consultations. Armed with this knowledge, student pharmacists can effectively aid in the early detection of eye disorders, make appropriate referrals when necessary, and help prevent vision loss.

Using home medical equipment, digital health devices, and user-friendly apps can improve patients’ quality of life. These tools help with medication schedules, physical therapy activities, and overall wellness, leading to better health outcomes and empowering patients to take control of their well-being (refer to Table 6). Integrating these technologies into the self-care curriculum can help student pharmacists stay updated on the latest advancements in home health delivery and contribute to enhancing the quality of life for their patients.

It is common for many gastrointestinal (GI) disorders to go unnoticed until they are too advanced for effective treatment. Therefore, prompt self-care and timely referral can significantly improve patient outcomes. With digital health devices and applications on the market, there are now innovative ways to bridge this gap, allowing patients to manage their GI symptoms proactively through self-care. These advanced technologies encompass symptom tracking, nutritional guidance, medication adherence, telemedicine, and treatment plans (refer to Table 7).

Recognizing the significance of pediatric digital health technologies in monitoring children’s health is crucial. These technologies empower parents and caregivers to encourage healthy behaviors, facilitate medication adherence, provide patient education, and enable telemedicine. Pharmacy students will learn to help manage children’s health by gaining knowledge in pediatric self-care and addressing the unique needs of pediatric patients, who may not always fully communicate their needs (refer to Table 8).

Digital health devices and apps for respiratory disorders integrate innovative technologies to help patients manage and monitor their conditions at home (refer to Table 9). These include symptom monitoring, medication adherence, peak flow measurement, and telehealth. Educating pharmacy students in self-care for respiratory disorders can assist in efficiently managing chronic respiratory diseases at home.

When selecting digital health products, it is important to consider that many devices require a purchase. Most products are FDA cleared; some have undergone validation or other tests to prove their accuracy and feasibility. For example, the QardioArm^®^ device for monitoring blood pressure (Table 2) underwent a validation study, received FDA clearance, and is also eligible for FSA/HSA. These tests and verifications bolster the credibility of the product. Several other products, such as Omron Evolv^®^, Oura Ring^®^, ReCIVA^®^, MyndMove^®^, KinetiSense^®^, and OrCam MyEye^®^, have also undergone validation studies, demonstrating their ability to produce accurate data and clinical effects, ultimately enhancing the trustworthiness of these products [42,43,44,45,46,47,48]. 

#### 4.2.2. Population-Based Digital Health Education

Many devices and apps are valuable tools for individuals with specific health conditions who use these products for self-care. For instance, a blood pressure monitor, or EKG device can assist patients with heart diseases by monitoring their blood pressure daily and detecting any anomalies in their heart rate that require further medical attention. Additionally, products like KinetiSense^®^ (Table 6), a motion sensor, can detect and record the movements of patients with movement disorders for their self-care. A study has demonstrated that this motion sensor can identify irregularities in patients’ movements during medication wear-off, enabling pharmacists and clinicians to make precise adjustments to medication dosages. Moreover, apps like “Gratitude Affirmations” or “Ten Percent Happier” can support patients experiencing depressive moods, improving their mental well-being. 

In self-care, developing patient communication topics around case discussions involving patients who could use digital health devices and applications is beneficial. Surprisingly, a survey revealed that less than half of pharmacy schools currently include digital health education in their curriculum [11]. Furthermore, the extent of coverage varies widely, with some schools providing just one to two lectures per academic year and others offering more comprehensive modules or courses. This indicates a significant educational gap that needs to be addressed in digital health.

#### 4.2.3. Challenges with Incorporating Digital Health in Self-Care Pharmacy Education and Potential Solutions

Education is crucial in developing students’ competencies and enhancing patient care. However, integrating topics related to digital health into the pharmacy curriculum poses notable challenges. 

One significant challenge is the lack of standardized approaches for incorporating digital health into existing curricula. The wide variety of digital devices and applications, coupled with the fast pace of technological advancement, presents a challenge for pharmacy education to stay abreast of the latest innovations. Additionally, the diverse backgrounds of pharmacy students may result in varying levels of understanding of digital technologies. These factors contribute to the significant challenge of creating tailored educational experiences that ensure student engagement and practical learning.

Efforts to integrate digital health into pharmacy curricula date back to 2016. One study outlined a well-structured educational intervention designed to train student pharmacists in navigating this field. The program included a web-based lecture and an interactive workshop in which students were taught how to find, evaluate, and utilize medical apps. At the end of the course, students reported significant improvements in their ability to find, evaluate, and use medical apps in patient care. They also expressed high satisfaction with the learning experience [49]. 

When considering curriculum integration, it is important to consider the logistics of incorporating digital health into self-care courses and other parts of the pharmacy curriculum. It is crucial to avoid overwhelming students and to maintain the focus on core training. A recent study, in 2023, suggested strategies for integrating digital health throughout the pharmacy curriculum, examining the impact of this integration over time. The intervention took place over an academic year. It involved integrating digital health concepts into an existing required course—a weekly case-based, discussion-oriented conference series alongside therapeutic courses. Students were given a case and prework related to digital health topics each week before the interactive learning session. The digital health topics discussed included wearable health technology, mobile health apps, sensor-enabled medication devices, telehealth, and electronic health records. The discussion-oriented nature of the case conference made it possible to incorporate digital health without adding extra teaching hours to the curriculum and encouraged active student participation. Overall, the intervention significantly increased students’ familiarity, comfort, and knowledge regarding digital health [50]. This approach can serve as a model for other pharmacy programs. 

Another obstacle is the limited number of qualified faculty with expertise in digital health. There is an evident shortage of faculty with the experience and knowledge to advance digital health education on a larger, formal scale. Half of the faculty surveyed identified the lack of experts as the primary obstacle to integrating digital health into the pharmacy curriculum [11]. To overcome this challenge, developing train-the-trainers programs focused on equipping and supporting faculty with the necessary skills and knowledge in digital health is essential. This will enable them to effectively teach and integrate digital health topics into the existing curriculum.

One other challenge we face is managing the allocation of class lecture time. For instance, while most current digital health products and applications focus on self-care wellness screening, insufficient class time is available to delve deeply into this aspect of digital health. Our self-care wellness screening module consists of five hours of didactic instruction, four hours of practical training, and a one-hour hands-on assessment, totaling ten hours of class time. Therefore, as mentioned earlier, the most effective learning strategies, without increasing additional teaching hours, are likely to involve team-based peer-teaching, independent primer readings outside the classroom, case discussions, SOAP note application, role-playing, or OSCEs.

Moreover, pharmacy institutions can implement various approaches, such as interprofessional collaboration, practical application, and lifelong learning strategies. For example, as an interprofessional education opportunity, pharmacy students can collaborate with peers from other health disciplines to create posters or deliver presentations on topics related to digital health devices and applications. Using real-world scenarios, practical assessments can offer practical training on various digital health concepts. Lastly, fostering student pharmacists’ participation in emerging educational opportunities and professional networking can bolster their academic achievement, life-long learning, and career advancement.

### 4.3. Drawbacks of Digital Health

Integrating digital technologies into the field of digital health offers numerous advantages, but it also presents several drawbacks and challenges, particularly related to data policies and access to personal information. These challenges can be broadly categorized into privacy concerns, regulatory issues, data security, and digital literacy. Privacy concerns may involve data sensitivity and consent management, while regulatory issues may encompass compliance and approval processes. Data security concerns include cybersecurity threats and data integrity issues. Additionally, individuals may inevitably encounter challenges when learning to use digital health devices and applications. While digital health technologies have demonstrated their ability to enhance healthcare delivery and improve patient outcomes, it is important to consider these aformentioned drawbacks and challenges.

### 4.4. AI and the Future

In the era of digital health, integrating AI technology into self-care courses can provide students with a profound understanding of various digital health information and telehealth topics, which can be valuable in their future careers, especially in pharmacy. By introducing students to a wide array of digital health devices and applications early in their education, they can gain a comprehensive understanding of these tools and their potential in patient education, medication management, telehealth, and remote monitoring. AI-powered devices and tools can play a crucial role in helping pharmacy students acquire the skills and knowledge needed to excel as healthcare providers in the future.

This research focuses on topics pertinent to digital health in self-care education. As a future educational direction, educating student pharmacists about integrating data policies for digital health technologies is crucial. Understanding how data from these tools can support research and the development of new tools to alert patients and healthcare providers is also important. The curriculum should cover how artificial intelligence can assist patients by tracking and analyzing physical activities and disease progression, educating them about proper nutrition and changing unhealthy eating habits, creating personalized wellness programs, and providing virtual counseling.

### 4.5. Study Limitations

There are several limitations associated with integrating digital health into self-care courses. Firstly, since the concept of digital health is relatively new, limited research is available, leading to varying perspectives and potential biases. Furthermore, the broad and rapidly evolving nature of digital health, coupled with the wide range of applications and devices, makes it challenging to establish a gold standard for educational strategy. Realistically, we can only cover the digital health concepts most relevant to the self-care curriculum and may not be able to include all products. Lastly, it remains uncertain whether older users or individuals with poor health literacy will find the digital health products discussed in this paper easy to use or effective.

There is a need for standardized ratings and unified categorization for digital health devices or apps. The absence of such standards may result in a biased selection of these devices or apps. Furthermore, the limited number of volunteers conducting our validations may also introduce some level of bias.

In our research, our primary goal was to pinpoint potential applications and devices to be included in pharmacy self-care courses. However, we could not conduct practical pilot testing of these products because of financial constraints. We are aware of this limitation and suggest that future studies involve hands-on testing of these applications and devices once funding is secured to obtain the necessary products.

## 5. Conclusions

In summary, it is crucial to identify pertinent digital health topics for inclusion in self-care courses at pharmacy schools to equip student pharmacists with the necessary skills for the rapidly advancing field of digital health. Integrating these topics will enhance their capacity to deliver exceptional, patient-centered care and prepare them for emerging self-care technologies.

**Figure 2:**
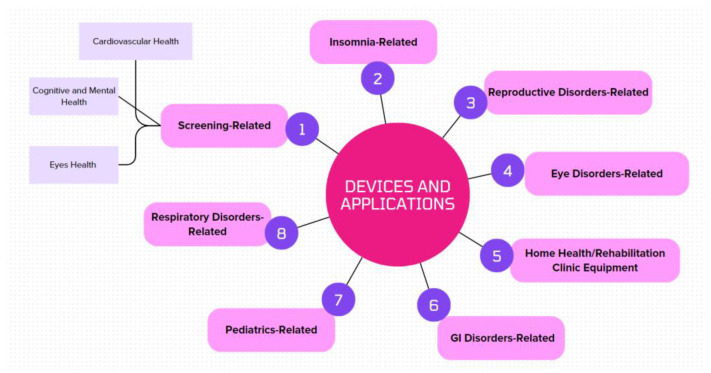
Summary of all self-care-related devices and applications.

The devices and applications discussed in this research are examples that can be integrated into a pharmacy self-care curriculum, covering various health and wellness areas such as insomnia, gastrointestinal disorders, reproductive disorders, eye disorders, pediatrics, respiratory disorders, and home medical equipment and applications (refer to Figure 2). These topics are typically covered in introductory pharmacy school courses focusing on self-care. It is important to note that while most available devices and applications are related to cardiovascular health and wellness screenings, there are also valuable tools for improving sleep quality, addressing fertility and menstrual issues, maintaining eye health, and early detection of gastrointestinal disorders.

Most of these devices and applications are user-friendly and easy to operate. They can be utilized across different disease topics and age groups. As discussed in Section 4.2.1 and  Section 4.2.2, most of these devices are FDA cleared, and some have undergone validation or randomized controlled trial studies to demonstrate their accuracy, efficacy, and feasibility, such as Omron Evolv^®^, QardioArm^®^; Oura Ring^®^, ReCIVA^®^, MyndMove^®^, KinetiSense^®^, and OrCam MyEye^®^.

In light of the diverse range of devices and applications, each offering unique functionality and complexity, combined with the rapid pace of technological advancement and the lack of faculty experts, lecture hours, and standardized guidelines for integrating digital health into pharmacy education, there are significant challenges to implementing these important topics in the current curriculum. To address these challenges, developing strategies for creating tailored educational experiences, promoting student engagement and effective learning, and staying current with health technologies is crucial.

While digital health use is becoming popular, some limitations prevent it from being a perfect tool for self-care. Concerns about personal information and data security, as discussed in Section 4.3, persist. In addition, digital literacy could challenge specific populations in adapting to this new and rapidly evolving concept. Given the broad nature of self-care, establishing a universally applicable standard for devices and apps to guarantee accuracy and effectiveness may be a formidable task.

Our review encompassed many digital health devices and apps in self-care and their relevance for patient use across different disease states. It is important to note that our research did not specifically focus on practical implications. This sets the stage for future researchers to explore how effectively patients utilize digital health devices and apps in real-world settings and how this relates to pharmacy curriculum coverage.

Furthermore, as discussed in Section 4.4, future research in digital health may include artificial intelligence, telemedicine, portable technology, digital therapeutics, and data security. As digital health technology continues to improve, ongoing research is needed to develop the best strategies for incorporating relevant digital health in self-care education and define the best student-learning strategies. 

## Figures and Tables

**Table 1:** Summary of the self-care-related digital health validation results.

Devices/Apps	Technological Relevance to Self-Care, i.e., Is This Product/App Self-Care Related (Yes = 1 Point; No = 0 Point)	Compatibility with Existing Pharmacy Self-Care Curricula (Yes = 1 Point; No = 0 Point)	Evidence of Educational Enhancement, Either Student Pharmacists, or Patient/Caregivers (Yes = 1 Point; No = 0 Point)	Overall Scores (Total Scores = 39)
KardiaPro	13	12	13	38
OMRON Evolv	13	13	13	39
Qardio Arm	13	13	13	39
Kardia Mobile 6L	12	11	13	36
ASCVD	11	11	13	35
Livongo	13	13	13	39
Lumosity	12	7	11	30
Gratitude Journal, Affirmation	11	7	10	28
Ten Percent Happier	12	7	11	30
My Health Record	13	12	11	36
Omada Prevent Digital	13	13	13	39
Health	13	13	12	38
Neurotrack	10	6	12	28
Eye Sync	10	11	11	32
ISense SMART Adjustable	13	13	13	39
Oura Ring	13	13	13	39
Sleepio	13	13	12	38
Contraception App	13	13	12	38
Evvy Vaginal Health	12	12	13	37
OvuSense OvuCore	13	13	12	38
OrCam MyEye	13	11	13	37
Kinetisense 360	8	7	11	26
MyndMove	8	5	11	24
Breath biopsy ReCIVA Breath Sampler	11	5	12	28
My GI Health	13	13	12	38
Sound Scouts	13	13	13	39
GoCheck Kids	13	13	11	37
SmokeFree	13	12	13	38
WearOptimo Micro Wearable Devices	13	9	12	34

**Table 2:** Screening-related devices and applications.

Devices/Apps	User Friendliness	Features	Take-Home Message	Misc.	Ease-of-Use Scale 1—Extremely Easy 2—Moderately Easy 3—Least Easy/Requires Study
Cardiovascular Health					
KardiaPro Kardia Mobile 6L [12] https://alivecor.com accessed on 3 March 2024. - Irregular heartbeat and blood pressure monitoring devices	These devices can monitor EKG, A-Fib, irregular heartbeat, or blood pressure. Different devices have slightly different functions. They can be connected to either the Kardia mobile app or used as a web-based app. These data can provide clinicians/pharmacists with information about a patient’s cardiac function and/or blood pressure.	KardiaPro: Clinicians/pharmacists can virtually monitor their patients whenever needed. Kardia Mobile 6L: It is able to record EKG, bradycardia, tachycardia, and other atrial fibrillation measurements significant to the patient.	Wearable Portable Mobile App Kardia Mobile 6L: Six-lead individual EKG	Available for iOS, Android, and web FDA cleared CE mark Kardia Mobile 6L: First portable 6L EKG device	2
OMRON Evolv [13] https://omronhealthcare.com/ accessed on 3 March 2024. - Blood pressure monitoring device	Attaches to patients’ smartphones and sends blood pressure numbers directly to their phones with the OMRON app. The device has a feature that allows patients to enter their blood pressure numbers manually.	Omron Evolv makes it easier for patients to check their blood pressure daily. It is portable and convenient and provides an accurate representation of blood pressure numbers to patients.	Portable Wireless/wired Can send results to clinicians	Available for iOS and Android FDA cleared CE mark	2
Qardio Arm [14] https://www.qardio.com/ accessed on 3 March 2024. - Blood pressure monitoring and irregular heartbeat detection device	Qardio Arm smart blood pressure monitor is not only portable but wireless as well, which makes it easy for patients to use.	Qardio Arm allows patients to also measure their irregular heartbeat.	Triple measurement	FDA cleared FSA/HSA eligible	1
ASCVD [15] https://cvquality.acc.org/clinical-toolkits/ascvd-risk-estimator accessed on 3 March 2024. - ASCVD risk calculator app	Simple interface to input data needed to calculate the ASCVD risk of a patient.	This can be used by patients or providers to input a patient’s personal information, such as blood pressure readings and cholesterol labs. It also characterizes 10-year ASCVD scores as “low risk”, “borderline risk”, “intermediate risk” or “high risk”.	Predicts and characterizes a patient’s 10-year ASCVD risk score	Available for iOS and Android Free app	1
Livongo [16] https://www.livongo.com/ accessed on 3 March 2024. - Diabetes monitoring device	These devices monitor, store, and upload data to the corresponding apps automatically for a patient. Livongo for Diabetes allows patients to reorder lancets and strips through the monitor without additional costs (unlimited test strips).	It has 24/7 coaching, and the program can be personalized. The device provides support immediately after an out-of-range device reading. The Livongo for Diabetes blood glucose monitor offers automatic data uploading. The Livongo for Hypertension blood pressure monitor records readings in the mobile app.	Provides coaching and personalized strategies to patients diagnosed with T1DM, T2DM, or HTN Allows for tracking readings with Livongo smart devices	FDA cleared	3
Omada Digital Care Platform [17] https://www.omadahealth.com/platform accessed on 3 March 2024. - Health coaching program	Omada provides a cellular-connected device that automatically syncs readings to the app for progress tracking.	It offers virtual care programs targeting prediabetes, diabetes, hypertension, and musculoskeletal conditions with personalized plans, health expert support, and coaching, such as in nutrition guidance, lifestyle management, and physical therapy.	Human-led digital care team that provides personalized plans for patients	FDA cleared	1
Cognitive and Mental Health					
Lumosity [18] https://www.lumosity.com/ accessed on 3 March 2024. - Brain health app/website	This application provides tutorials for its “brain games” to test a person’s cognition and allows users to choose which games they wish to play.	A cognitive training app that builds fun, interactive games for the user to challenge and improve their cognition. This app focuses on working to develop memory, speed, and problem-solving skills through puzzles, memory games, logic problems, and meditation techniques.	Mobile application Goal is to improve cognitive function	Free app	2
Neurotrack [19] https://neurotrack.com/ accessed on 3 March 2024. - Early dementia detection device	Easy-to-use device that does not require training and has an accessible design that uses symbols and numbers.	It helps with early diagnosis of dementia by completing a rapid 3 min screening that provides a score which can be used in healthcare clinics via a tablet or at home.	Early dementia diagnosis leads to better outcomes and cost reduction for health systems	FDA Class II device	2
Gratitude Journal, Affirmation [20] https://blog.gratefulness.me/gratitude-affirmations/amp accessed on 3 March 2024. - Mental health strengthening app	This mobile app sends daily prompts through notifications to help the user journal and maintain a habit of journaling.	It provides positive affirmations to develop a healthy routine and self-image. The “Daily Zen” feature allows the user to see other people’s journeys toward mindfulness and gratitude.	Helps the user focus on their mental health	Available for iOS	1
Ten Percent Happier [21] https://www.tenpercent.com/ accessed on 11 March 2024. - Mental health strengthening app/website	Meditations are guided and easy to view and take approximately 10 min.	Uses guided meditations, videos, talks, and sleep content to improve/increase meditation practices with the goal of improving relationships and sleep habits and becoming more mindful and happier overall.	Meditation sessions, videos, and articles are used to promote self-care and healthy habits and outlooks on life	Free trial for 7 days, but there is a cost for membership after the trial period ends	1
Health Records					
My Health Record [8] https://www.digitalhealth.gov.au/ accessed on 11 March 2024. - Medical records management app/website	Summaries and results from a patient’s recent visit can be easily viewed through their mobile phone.	A mobile application and website that lets patients upload and keep track of documents related to their health.	Can track family members’ health Finds the nearest health service Tracks immunizations, allergies and reactions, and care-planning documents	Available for iOS, Android, and web	2
Apple Health [22] https://www.apple.com/ios/health/ accessed on 11 March 2024. - Comprehensive health app	This app is easy to navigate, as it categorizes readings and provides alerts when there is a new trend in one’s health data.	Tracks daily activities (steps, workout activity, and sleep habits). Allows patients to schedule and track interactions of their medications. The app can be paired with an Apple iWatch for more features. The health data can be shared with providers and can provide first responders with critical health information in an emergency.	Allows users to track their daily activity and health and share these data with their providers	Free app Predownloaded on Apple iPhones and watches	1
Eye Health					
Eye Sync [23] https://www.neurosync.health accessed on 11 March 2024. - Eye-related cognitive function detection device	Users receive initial training and onboarding by NeuroSync experts to use and interpret results.	Data are received within a minute from a wireless and portable device. The results can be exported via PDF or transferred to an EMR.	A portable device that measures the synchronization of eye movement to represent cognitive function	Cleared by FDA in 2016 for visual impairment identification Cleared by the FDA in 2019 as a Breakthrough Device Designation for aid in concussion assessment Cleared by FDA in 2021 as an aid in concussion diagnosis	2

EKG: electrocardiography; FDA: Food and Drug Administration; CE mark: Conformité Européenne mark; FSA: flexible spending account; HSA: health saving account; 6L: six leads; T1DM: type 1 diabetes mellitus; T2DM: type 2 diabetes mellitus; HTN: hypertension; ASCVD: atherosclerotic cardiovascular disease; EMR: electronic Medical Record.

**Table 3:** Insomnia-related devices and applications.

Devices/Apps	User Friendliness	Features	Take-Home Message	Misc.	Ease-of-Use Scale 1—Extremely Easy 2—Moderately Easy 3—Least Easy/Requires Study
ISense SMART Adjustable [24] https://www.myisense.com/ accessed on 3 March 2024. - Sleep score and sleep heart-rate tracking device	ISense SMART Adjustable tracks a patient’s heart rate, respiratory rate, sleep movements, sleep cycles, and overall sleep score.	The device uses a specific design to create a pillow with filling that does not move while the patient is sleeping.	Has built-in sensors that are connected to the application on an iPhone or Android to track the patient’s overall sleep score	Available for iOS and Android	3
Oura Ring [25] https://ouraring.com/ accessed on 3 March 2024. - Sleep score tracking device	Oura Ring tracks a patient’s sleep activity by wearing the ring on their finger at night.	The Oura Ring was created to provide patients with accurate information, since it can measure sleep with the palm of their finger, which has a pulse that is strong enough to read.	Continuous monitoring	Lifestyle and wellness	2
Sleepio [26] https://www.sleepio.com/ accessed on 3 March 2024. - Sleep improvement program	Sleepio is a sleep improvement program that takes 6 weeks, including the use of cognitive and behavioral therapy techniques.	Sleepio allows patients to fall asleep quicker and stay asleep throughout the night.	Takes 20 minutes a day for 6 weeks online, with sleep experts communicating different sleeping techniques with patients	Available for iOS, Android, and web	1

**Table 4:** Reproductive disorder-related devices and applications.

Devices/Apps	User Friendliness	Features	Take-Home Message	Misc.	Ease-of-Use Scale 1—Extremely Easy 2—Moderately Easy 3—Least Easy/Require Study
Contraception App [27] https://www.cdc.gov/reproductive-health/hcp/contraception-guidance/app.html accessed on 3 March 2024. - Contraception guideline app	The Contraception App is geared toward healthcare professionals as an extra resource to be used in their practice and learning experiences.	The application compiles information from Reproductive Access Project (RHAP), CDC, FDA prescribing information, and other sources to provide information and guidance for contraceptive care for women.	Easily accessible informational guide for clinicians and trainees	FDA cleared	2
Evvy Vaginal Health [28] https://www.evvy.com/ accessed on 12 March 2024. - Vaginal health product	Evvy Vaginal Health contains an at-home swab test that is sent back to the facility for analysis and results.	Women can monitor vaginal health to prevent future infection and add an STI test. A subscription lets women have a consultation with a doctor and receive prescription treatment.	A home testing kit to evaluate a woman’s vaginal health and a treatment program, if needed, based on results	FDA cleared	2
OvuSense OvuCore [29] https://www.ovusense.com/ accessed on 12 March 2024. - Ovulation and PCOS monitor device	Data from sensors are easily transferable to a smartphone app.	Tracks ovulation so that a patient may successfully get pregnant. A subscription allows for a nurse consultation and the doctor to monitor issues.	An accurate menstrual cycle monitor to track ovulation dates for fertility purposes, especially helpful for those who have irregular cycles	FDA-approved and class II medical device The sensor must be cleaned at first use and before and after each use Subscription-based	1

CDC: The Centers for Disease Control and Prevention; FDA: Food and Drug Administration; STI: sexually transmitted infection; PCOS: polycystic ovary syndrome.

**Table 5:** Eye disorder-related devices and applications.

Devices/Apps	User Friendliness	Features	Take-Home Message	Misc.	Ease-of-Use Scale 1—Extremely Easy 2—Moderately Easy 3—Least Easy/Requires Study
OrCam MyEye [30] https://www.orcam.com/en-us/home accessed on 3 March 2024. - Vision aid device.	OrCam attaches virtually to a pair of glasses and allows patients to see better.	The device can read the text of a book or an electronic device to patients.	Wearable	OrCam MyEye give patients visual information audibly	1
ClearView-360 [31] https://www.ophtha lytics.com accessed on 3 March 2024. - Vision aid software	ClearView-360 software allows patients to perform quick screenings for signs of diabetic retinopathy and age-related macular degeneration.	ClearView-360 is a software that gives patients information about diabetic retinopathy by telling them how severe the disease is with a report.	ClearView-360 software provides patients with a detailed report allowing clinicians/pharmacists to develop a comprehensive treatment plan	Class II medical device FDA presub for the de novo route	2

FDA: Food and Drug Administration.

**Table 6:** Home health/rehabilitation clinic equipment.

Devices/Apps	User Friendliness	Features	Take-Home Message	Misc.	Ease-of-Use Scale 1—Extremely Easy 2—Moderately Easy 3—Least Easy/Requires Study
Kinetisense 360 [32] https://www.kinetisense.com/ accessed on 3 March 2024. - Motion capture analysis system device	Kinetisense 360 has a customizable interface for practitioners.	Kinetisense 360 is a portable, single-sensor device that automatically reports data. It can track trends and progression in the patient’s data. Each assessment takes 5 seconds.	Allows patients and providers to have a visual, objective representation of improvements, which leads to better adherence to physical therapy/treatment	Not FDA cleared	3
MyndMove [33] https://www.neuphysio.com/myndmove/ accessed on 3 March 2024. - Functional electrical stimulation device	MyndMove is used specifically within clinics.	This device allows for the rehabilitation of patients and gives them independence once treated. It is an 8-channel stimulator that can stimulate up to 8 different muscle groups.	Electrodes are used to help patients regain upper body function	Noninvasive therapy	2

FDA: Food and Drug Administration.

**Table 7:** GI Disorder-related devices and applications.

Devices/Apps	User Friendliness	Features	Take-Home Message	Misc.	Ease-of-Use Scale 1—Extremely Easy 2—Moderately Easy 3—Least Easy/Requires Study
Breath biopsy ReCIVA Breath Sampler [34] https://www.owlstonemedical.com/ accessed on 11 March 2024. - Breathalyzer for disease detection device	User-friendly and compatible with home use; in the future, it can be used by anyone without specialized training.	Noninvasive procedure for early detection of various diseases, such as inflammatory, respiratory, liver, metabolic, CV, ID, and cancers.	Only used for clinical and research purposes	Works with their other products, such as CASPER Portable Air Supply, used to filter out contaminants, and Breath Biopsy Collect software, used to collect data	2
My GiHealth [35] https://mygi.health/app accessed on 06 March 2024. - GI health app	My GiHealth shows easy to read charts to track symptoms.	Provides lifestyle, diet, and medication recommendations to improve GI disorders including IBS, constipation, diarrhea, and GERD.	Allows patients with GI disorders to track their symptoms and improvements and make further lifestyle changes to enhance their treatment plan	FDA cleared Available for iOS	1

FDA: Food and Drug Administration; CV: cardiovascular disease; ID: infectious disease; GI: gastrointestinal; GERD: gastroesophageal reflux disease; IBS: irritable bowel syndrome.

**Table 8:** Pediatric-related devices and applications.

Devices/Apps	User Friendliness	Features	Take-Home Message	Misc.	Ease of Use Scale 1—Extremely Easy 2—Moderately Easy 3—Least Easy/Require Study
Sound Scouts [36] https://www.soundscouts.com/ accessed on 11 March 2024. - Hearing test app	The testing and results can all be seen through a tablet or mobile phone.	It is inexpensive ($23) and includes 4 test sessions with 3 separate hearing tests for those 4 years old and up. Clinicians are able to purchase in bulk for patients to claim in a discounted price. Children can be supervised by adults.	Series of games that test children’s hearing to check for 3 hearing issues: conductive hearing loss, sensorineural hearing loss, and difficulties listening to noise The 3 tests include a test of speech under quiet conditions, a tone test, and a test of speech under noise conditions	Available for iOS and Android Equipment needed: smartphone/tablet and good quality headphones A quiet place is needed for tests	2
GoCheck Kids [37] https://www.gocheckkids.com/ accessed on 11 March 2024. - Eye-related health app	An easy-to-use smartphone app that can take a picture of a child’s nondilated eye, which can determine which children are at risk of amblyopia (i.e., lazy eye).	It is a noninvasive procedure that can detect early vision impairment and does not require children to read off of visual acuity test charts if they are not able to read yet.	Eye screening through a smartphone which can provide results and detect risk factors for vision impairment immediately to save time	Available for iOS	2

**Table 9:** Respiratory disorder-related devices and applications.

Devices/Apps	User Friendliness	Features	Take-Home Message	Misc.	Ease-of-Scale 1—Extremely Easy 2—Moderately Easy 3—Least Easy/Requires Study
SmokeFree [38] https://smokefreeapp.com/ accessed on 3 March 2024. - Smoking cessation app	SmokeFree is an easy-to-navigate app that allows users to view their smoke-free time in their dashboard easily. It allows users to chat with advisors/coaches and maintain a diary in which they can record their progress in terms of cravings. It also provides online clinics for users to attend to motivate them further.	Provides motivational phrases and tips for smoking cessation and provides a visual representation of a patient’s journey. If a person needs to change the date of when they quit smoking, the app is supportive and helps celebrate wins by giving badges whenever the user achieves a goal.	Motivates users in smoking cessation	Available for iOS and Android Has notification alarms to remind users to use the app	2
WearOptimo Micro Wearable Devices [39] https://thehealthhorizon.com/showcases/innovations/4b3ae5d3-22a6-4a97-aef8-d5397da8532c accessed on 03 Mar 2024. - Wearable sensor for detecting respiratory failure	A wearable sensor that can detect respiratory failure in patients with COVID-19. It can also potentially replace blood tests for diseases needing frequent monitoring of blood counts.	Provides early detection of respiratory failure through skin penetration to enable continuous monitoring of IL-6 levels in patients with COVID-19.	The sensor will be applied to the skin to detect the development of serious diseases, which can help providers care for patients immediately	A patent has been acquired, but the sensor is still a work in progress	2

IL-6: interleukin 6.

## Data Availability

Information regarding products and applications was retrieved from publicly available information on the manufacturers’ websites or the Google App Store.
